# Defluoridation technology for drinking water and tea by green synthesized Fe_3_O_4_/Al_2_O_3_ nanoparticles coated polyurethane foams for rural communities

**DOI:** 10.1038/s41598-017-08594-7

**Published:** 2017-08-14

**Authors:** Sonu Kumari, Suphiya Khan

**Affiliations:** Department of Bioscience and Biotechnology, Banasthali Vidyapith, Banasthali Tonk, Rajasthan, 304022 India

## Abstract

Fluoride (F) contaminated ground water poses a serious public health concern to rural population with unaffordable purification technologies. Therefore, development of a cost-effective, portable, environment and user-friendly defluoridation technique is imperative. In the present study, we report on the development of a green and cost-effective method that utilizes Fe_3_O_4_ and Al_2_O_3_ nanoparticles (NPs) that were synthesized using jojoba defatted meal. These NPs were impregnated on to polyurethane foam (PUF) and made into tea infusion bags. The Al_2_O_3_ NPs-PUF displayed a higher water defluoridation capacity of 43.47 mg g^−1^ of F as compared to 34.48 mg g^−1^ of F with Fe_3_O_4_ NPs-PUF. The synthesized Al_2_O_3_-PUF infusion bags removed the F that was under the permissible limit of 1.5 mg L^−1^. The sorption experiments were conducted to verify the effect of different parameters such as pH, contact time, size of PUF and initial F concentration. The different properties of adsorbent were characterized using a combination of FESEM, EDX, XRD and FTIR techniques, respectively. The calculated total cost per NPs-PUF pouch developed is as low as US $0.05, which makes the technology most suitable for rural communities. This paper will be beneficial for researchers working toward further improvement in water purification technologies.

## Introduction

Water scarcity is considered as a major crisis of the 21^st^ century. It is reported that in 2015 approximately 663 million people lack access to safe drinking water worldwide^1^. Fluoride (F) is a major contributor to the world water crisis, affecting about 200 million people worldwide. It is reported that around 24 countries are severely affected by high F concentration in drinking water^2^. The rural population is more prone to F contamination as in some places, the available techniques are neither acquainted nor affordable. The fluorosis is reported more prevalent in rural population due to excess F contaminated water inevitably consumed by the rural population^3, 4^. F is known to cause mottled enamel, osteoporosis, crippling skeletal fluorosis, thyroid imbalance, growth retardation, kidney imbalance, types of morbidity and in severe cases leading to mortality^5^. Several methods have been developed to efficiently remove F from water, including nanofiltration, reverse osmosis (RO), coagulation, electrocoagulation, electrochemical oxidation, ion exchange and adsorption^6–9^. Till date, the defluorinated water at the community level in the outreach areas is far away due to its high cost and complex treatment modalities.

After water, tea infusions are the most popular beverages consumed worldwide by communities^10^. It is well-known that tea plants can accumulate F, for example, in 1930 it is reported that *Camellia sinensis* (tea plant) is a hyperaccumulator plant of F^11^. F concentrations above permissible limit were reported in tea drinks of India (1.55–3.21 mg L^−1^), China (1.60–7.34 mg L^−1^), Kenya and Tibet (2.59 mg L^−1^)^12–14^. The cost and effectiveness of the defluoridation techniques are still not satisfactory and thus required further improvements.

Among the reported techniques, adsorption is considered more advantageous for the rural population as it is inexpensive, rapid, easy to operate and highly efficient^15^. Several traditional adsorbents were reported such as activated carbon, zeolites and bone char but nanostructural materials proved highly efficient for F removal because of their high surface-to-volume ratio^16–18^. Various techniques have been known for nanomaterials production, such as reverse micelles, microwave, electrochemical, nonelectrochemical and green synthesis technique^19–23^. For the synthesis of nanomaterials, green chemistry route proved beneficial as compared to the chemical methods in term of its cost-effectiveness, environment-friendly and scalable properties^24^. Yet another challenge the researchers are facing is the separation of NPs from suspension after adsorption is the fact that NPs suspension form fine colloids in aqueous solution. To solve this problem, several researchers have used nanomaterials impregnated on support matrices. Diverse studies have been conducted using nanomaterials support matrices such as poly (acrylic acid) (PAA), polyurethane (PU) and poly (vinyl alcohol) (PVA)^25^. Recently, polyurethane foams (PUF) has been widely utilized and found promising in various water filtration systems because of its outstanding features of high-temperature resistance, UV resistance, enhanced mechanical property, abrasion resistance, easy availability and low cost^26, 27^. The impregnation of nanomaterials on the matrices can be achieved through various processes, such as surface nucleation, blending and dip coating^25, 28^. Dip coating technique was proved to be more favorable for impregnation due to advantages of its low cost and easy handling.

Recently, Al_2_O_3_/bio-TiO_2_ nanocomposite (ABN) and Al_2_O_3_/bio-TiO_2_ nanocomposite impregnated into electrospun TPU nanofiber membrane (ABN/TPU-NFM) have been developed for defluoridation of water samples. The adsorption capability of the developed adsorbent reported was 1.9 mg g^−1^. However, the adsorption capacity of material developed was relatively low, expensive and uneasy and unsuitable for rural areas^29^.

Therefore, the present research for defluoridation is focused on the development of easily affordable technology for the rural population. Here, Jojoba (*Simmondsia chinensis*) seed meal was utilized for green synthesis of NPs. The seed meal was obtained as a waste byproduct during the oil extraction process which we utilized for NPs synthesis. None of the available literature focused on the F removal using Fe_3_O_4_ and Al_2_O_3_ NPs impregnated PUF. Also, very few reports are available on the F removal from tea infusions, there is requirement to design cost-effective defluoridation technology for tea infusions. Kinetic and isothermic parameters were illustrated in order to describe the F adsorption mechanism. The interactions between F ions and adsorbent were analyzed by a combination of physico-chemical methods, such as field emission scanning electron microscopy (FESEM), energy dispersive X-ray (EDX) and Fourier transformed infrared (FTIR). Based on our experimental data, we demonstrated the successful F adsorption in samples and its mechanism through following three steps: (a) Green synthesis of Fe_3_O_4_ and Al_2_O_3_ NPs using novel defatted jojoba seed meal; (b) These NPs impregnated in polyurethane foams (PUF) and tested for defluoridation of water and tea samples; (c) Al-PUF tea infusion bag like pouches were found to be relatively more efficient in defluoridation of tea infusions. Overall, this work provides an inexpensive tool toward addressing public health and safety, especially at the resource-limited rural areas to mitigate health risks associated with ground water F contaminations.

## Results and Discussion

### Morphology and Chemical composition

**Figure 1:**
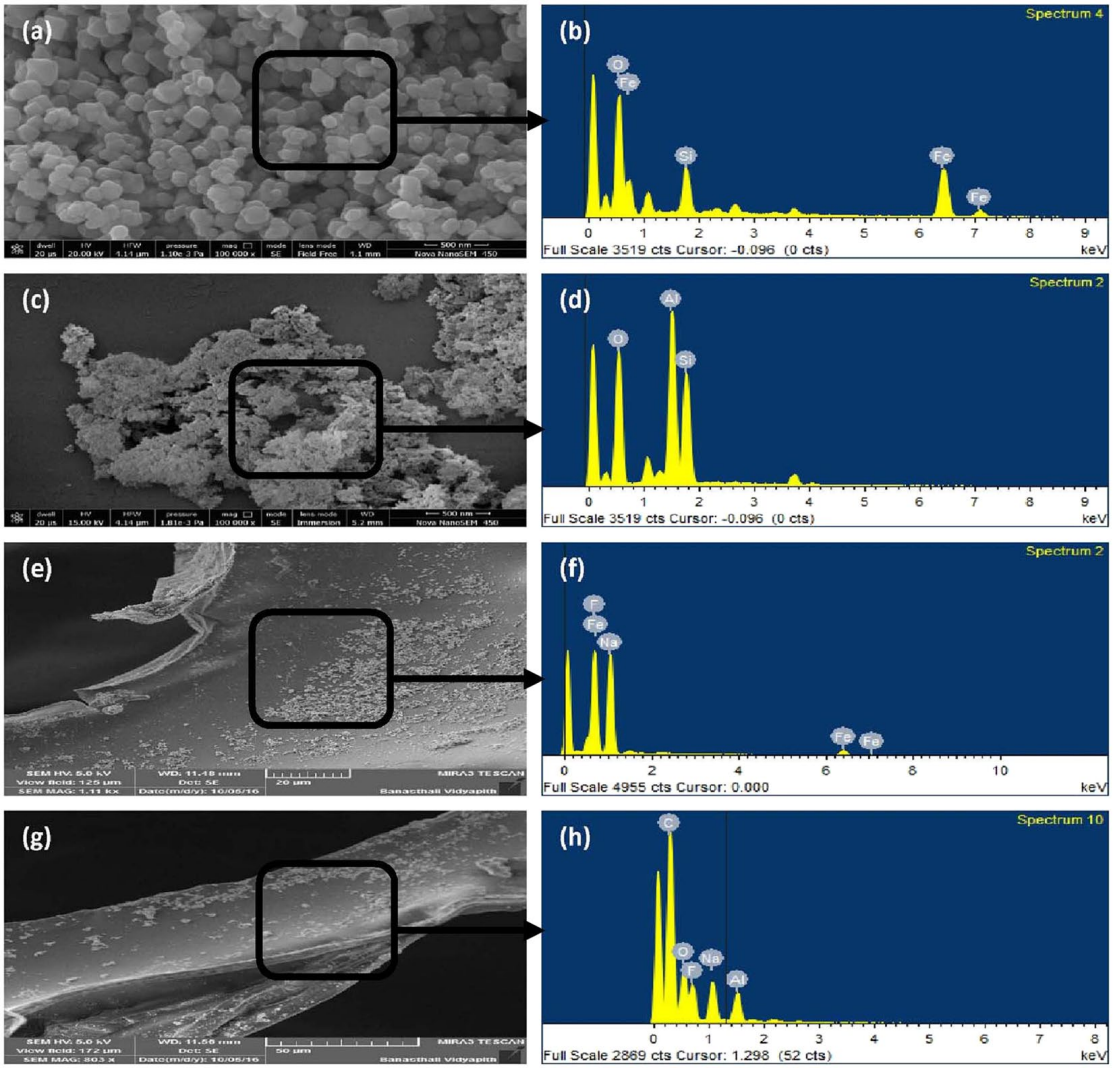
(**a**) and (**b**) show the FESEM image and EDX spectrum of Fe NPs, (**c**) and (**d**) show the FESEM image and EDX spectrum of Al NPs, (**e**) and (**f**) shows FESEM image and EDX spectrum of Fe NPs-PUF after F adsorption, and (**g**) and (**h**) show the FESEM image and EDX spectrum of Al NPs-PUF after F adsorption.

The Fe_3_O_4_ and Al_2_O_3_ NPs were synthesized from *Simmondsia chinensis* (jojoba) defatted meal extract by a green synthesis route as described in experimental methods. The surface morphology and elemental composition of synthesized Fe_3_O_4_ and Al_2_O_3_ NPs was characterized using FESEM and EDX spectrum analysis. Figure 1(a–d) clearly indicates the formation of Fe_3_O_4_ and Al_2_O_3_ NPs. The FESEM image illustrates spherical and rectangular shapes of green synthesized Fe_3_O_4_ NPs (Fig. 1a). The surface morphology of Al_2_O_3_ NPs appeared to be flakes in nature with irregular shape (Fig. 1c). The composition of NPs was further analyzed by EDX elemental mapping. The elemental composition from EDX analysis confirmed that the Fe_3_O_4_ NPs sample has O (36.99%), Fe (54.34%) and Si (8.66%) (Fig. 1b). Likewise, the EDX measurements with Al_2_O_3_ NPs showed the presence of O (53.11%), Al (25.09%) and Si (21.80%) (Fig. 1d). Based on the EDX spectrum analysis, it was confirmed the presence of both Fe and Al elements in the samples.

Figure 1(e and g) shows the FESEM images of PUFs after impregnation with Fe_3_O_4_ and Al_2_O_3_ NPs at different magnifications, indicating the binding of NPs. It is clear from the FESEM images that PUF has closed cell structure with NPs coated on its wall surfaces. The F peak in EDX spectrum showed the F adsorption process through Fe and Al NPs-PUF, confirming the F adsorption by an adsorbent (Fig. 1f and h).

**Figure 2:**
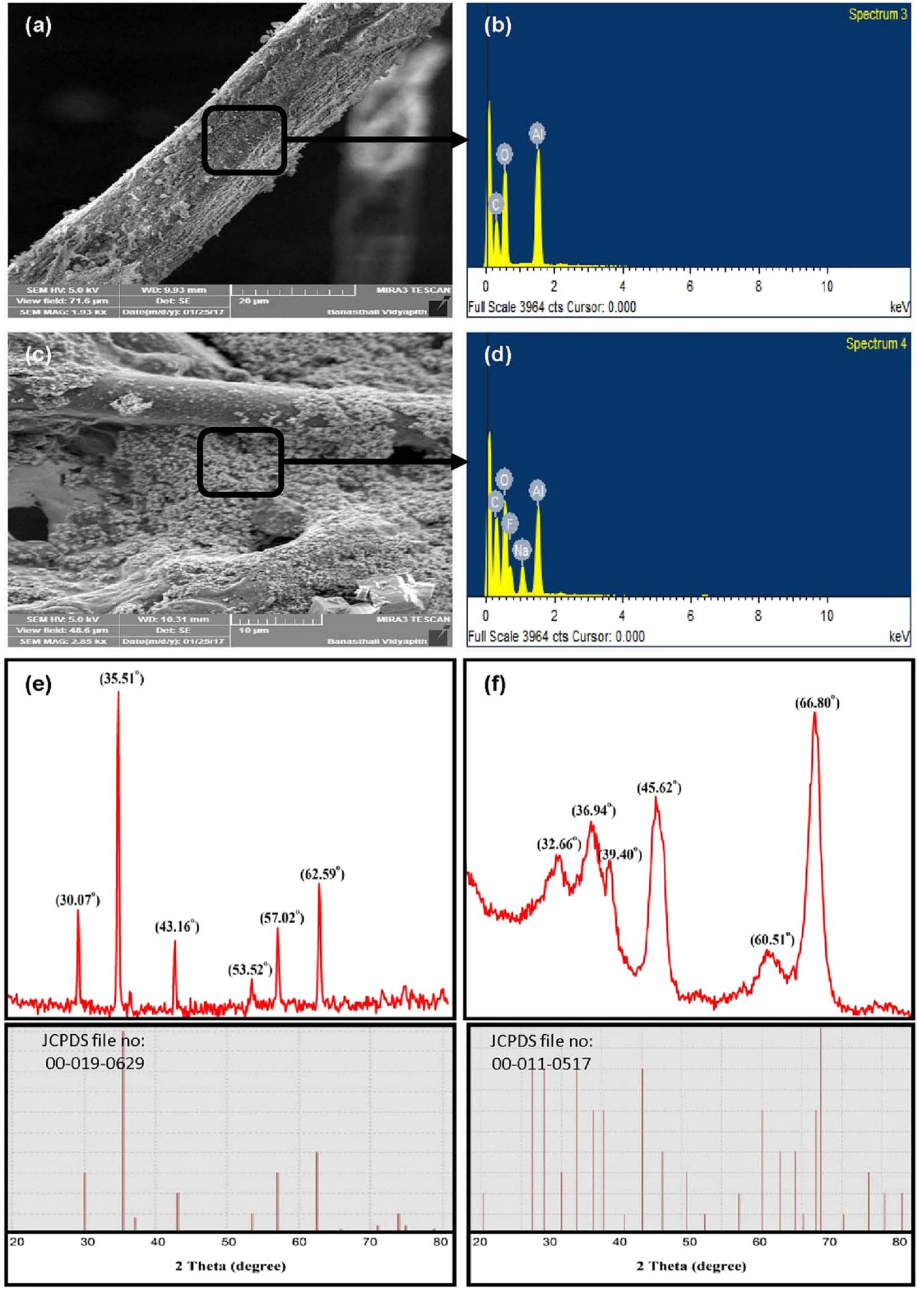
FESEM and EDX spectrum of (**a**) and (**b**) the Al-PUF tea bag, (**c**) and (**d**) the Al-PUF tea bag after F adsorption, and (**e**) and (**f**) shows XRD patterns of Fe_3_O_4_ NPs and Al_2_O_3_ NPs.

The tea bag covering filter paper surface morphology was studied by FESEM before and after F adsorption and is presented in Fig. 2(a and c). The FESEM analysis revealed that the Al-NPs were uniformly coated onto tea bag filter paper. The EDX spectrum confirmed the presence of Al element in Al-PUF tea bag sample before and after adsorption (Fig. 2b and d). The presence of F ions peak along with Al peak confirmed the F adsorption from tea infusions (Fig. 2d).

### Phase composition

Phase purity and crystallinity of the prepared Fe_3_O_4_ and Al_2_O_3_ NPs was recognized through XRD analysis. The XRD patterns of synthesized Fe_3_O_4_ and Al_2_O_3_ NPs are shown in Fig. 2(e and f). The three major diffraction peaks of synthesized Fe_3_O_4_ NPs were detected at 2θ = 35.51°, 62.59° and 30.07° (Fig. 2e), which are assigned to the crystal planes of (311), (440) and (220), respectively. The achieved peaks were similar to the standard patterns of JCPDS file no: 00-019-0629, which stated the crystallographic system of cubic structure of Fe_3_O_4_. For the Al_2_O_3_ NPs, intense diffraction peaks at 66.80°, 45.62° and 36.94° were observed, which corresponded to the planes (240), (−422) and (221), respectively (Fig. 2f). The XRD patterns declared the monoclinic crystal phase of Al_2_O_3_ NPs (00-011-0517). The average crystalline size of both NPs can be determined using the Debye-Scherrer equation^25^. The Debye-Scherrer equation:

1 $$D=\frac{k\lambda }{{\beta }_{hkl}\,{\cos }\,{\theta }_{hkl}},$$

where D is the crystallite size, k is Scherrer constant (0.9), λ is the X-ray wavelength of radiation for Cu Kα (0.154 nm), β_hkl_ is the full-width at half maximum (FWHM) and θ_hkl_ is the diffraction angle. The calculated average crystallite size of Fe_3_O_4_ and Al_2_O_3_ NPs was 51.48 nm and 11.64 nm. The XRD peaks observed similar to JCPDS file no: 00-019-0629 and 00-011-0517 confirmed the formation and structure of Fe_3_O_4_ and Al_2_O_3_ NPs. The Fe_3_O_4_ and Al_2_O_3_ NPs synthesized through the green method using defatted jojoba seed meal showed smaller size^30^.

### ζ-Potential and isoelectric point (IEP)

The surface charge of the Fe_3_O_4_ and Al_2_O_3_ NPs may have an important implication on their mobility and suspension stability in drinking water. The magnitude of surface potential decides the level of the electrostatic repulsion between particles. The *ζ*-potential of Fe_3_O_4_ and Al_2_O_3_ NPs is positive when pH is lower than the isoelectric point (IEP) of 7.1 and 8.7. Both NPs showed positive surface charges over a broad range of pH (2 to 9). The zeta-potential plot showed the moderate stability of Fe_3_O_4_ NPs and high stability of Al_2_O_3_ NPs.

### FTIR analysis

**Figure 3:**
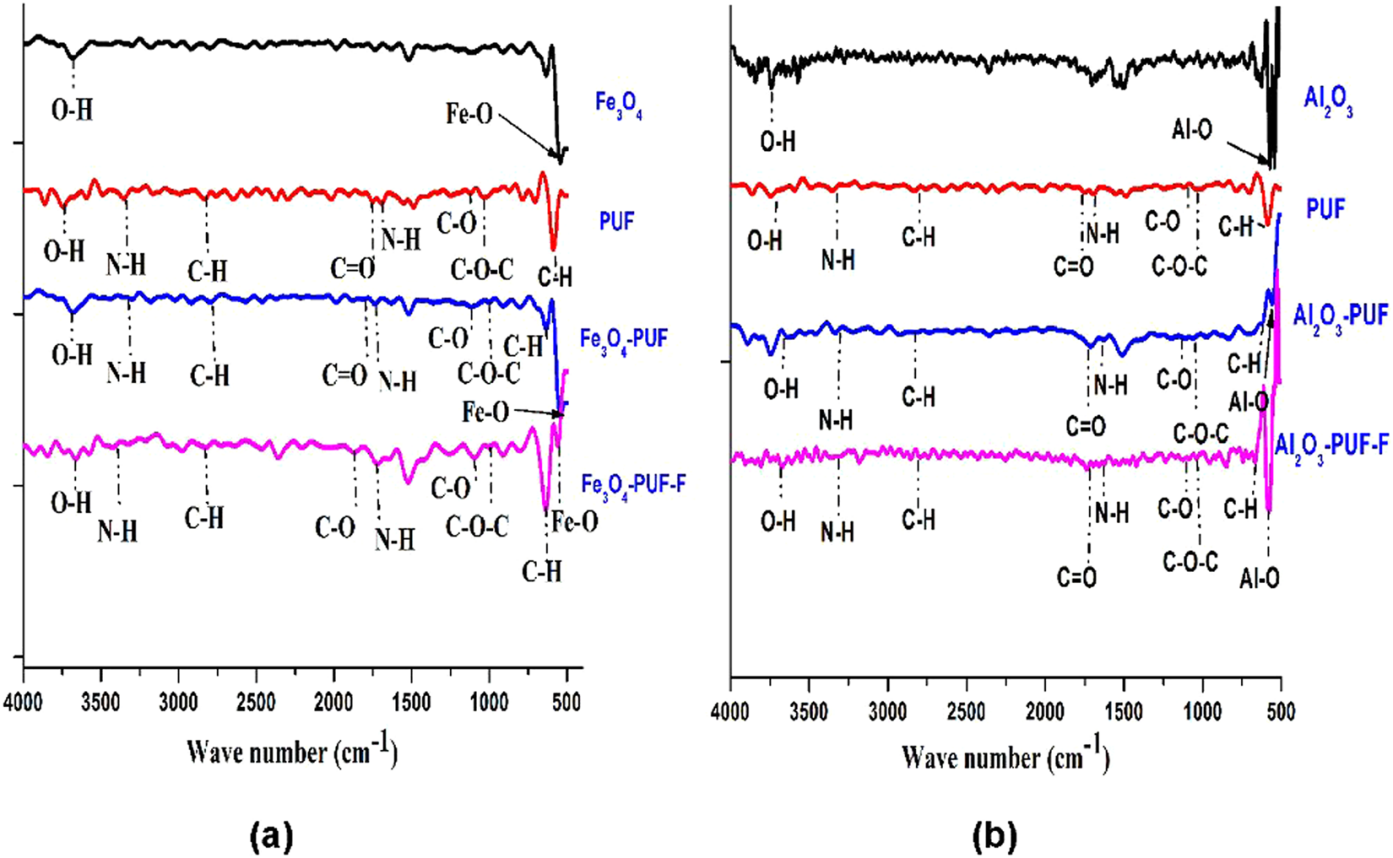
FTIR spectra of (**a**) Fe samples (**b**) Al samples.

The FTIR spectra of Fe_3_O_4_ NPs, PUF, and Fe_3_O_4_ NPs-PUF before and after F adsorption are presented in Fig. 3a. Characteristic peaks were observed at 556 and 3670 cm^−1^ in the spectra for Fe_3_O_4_ which are assigned to the stretching of metal-oxygen because of the Fe-O and O-H groups, respectively (Fig. 3a)^31^. The peaks occurred at 625, 1040, and 1120 in the PUF are assigned to the C-H, C-O-C stretch of ester and C-O stretch, respectively. The sharp peaks observed at 1640, 1730, 2840, 3300, and 3636 cm^−1^ in the PUF sample showed the presence of N-H stretch of urea, C=O stretch of urethane, C-H stretch, N-H stretch of urethane and urea, and O-H stretch, respectively (Fig. 3a). All the urethane functional group peaks were observed in PUF^32^. All the characteristic peaks observed in the Fe_3_O_4_ NPs and PUF were also observed in Fe_3_O_4_ NPs-PUF samples. The increased intensity of O-H and N-H band in Fe_3_O_4_ NPs-PUF spectrum before F adsorption indicated Fe chelation of N-H groups in PUF. The decreased intensity of Fe-O band after F adsorption can be associated with interaction with the F ions, the similar behavior of Fe-O was also reported in literature^33^ (Fig. 3a). The decrease in the intensity of O-H bond after F adsorption indicated the replacement of hydroxyl ions by the F ions^34^. The presence of Fe-O band in the spectra of Fe_3_O_4_ NPs-PUF before and after F adsorption confirmed that Fe_3_O_4_ NPs was complexed by PUF. The fact that no significant changes were observed in Fe_3_O_4_ NPs-PUF spectra before and after F adsorption showed that no significant structural changes occurred in the Fe_3_O_4_ NPs-PUF sample during the adsorption process.

The FTIR spectra of Al_2_O_3_, PUF, Al_2_O_3_ NPs-PUF before and after adsorption F from an aqueous sample are shown in Fig. 3b. The characteristic peaks observed at 574 and 3636 cm^−1^ in the Al_2_O_3_ sample are assigned to the stretching of metal-oxygen because of the Al-O and O-H group, respectively^35^. All the characteristic peaks of Al_2_O_3_ NPs and PUF were also detected in Al_2_O_3_ NPs-PUF samples, before and after adsorption of F. The intensity of Al-O peak decreased in the Al_2_O_3_ NPs-PUF bare sample that was possibly due to an interaction with PUF. Before F adsorption, the N-H and O-H band increased which shows the bonding of Al with O-H and N-H of PUF. However, after exposure with F, the intensity Al-O peak increased which was attributed to Al-O interaction with F ions and this result is consistent to the results previously reported in the literature^36^. Like in case of Fe NPs, Al NPs also showed the decrease in the intensity of O-H band after F adsorption was associated with the replacement of OH ions by F ions.

### Thermogravimetric analysis (TGA)

The thermal stability of the material before and after NPs impregnation was analyzed by TGA. The synthesized materials in this study are designed to be potentially used in water purification system and thus require the property to withstand a temperature range of 20–175 °C. The thermal stability of pure PUF, Fe_3_O_4_ and Al_2_O_3_ NPs-PUF, expressed in weight (mg) of the samples and temperature range was 30–700 °C. For the uncoated PUF, the initial thermal weight loss temperature recorded was 235.66 °C which moderately increased for Fe_3_O_4_ and Al_2_O_3_ NPs-PUF with ~249.66 and 247.33 °C, respectively. This result suggested the higher stability of Fe_3_O_4_ NPs-PUF than Al_2_O_3_ NPs-PUF which is consistent to a previously reported study by Alavi Nikje *et al*. showing delayed degradation process due to NPs impregnation on PUF^32^.

### Adsorption of Fluoride

#### Effect of pH

**Figure 4:**
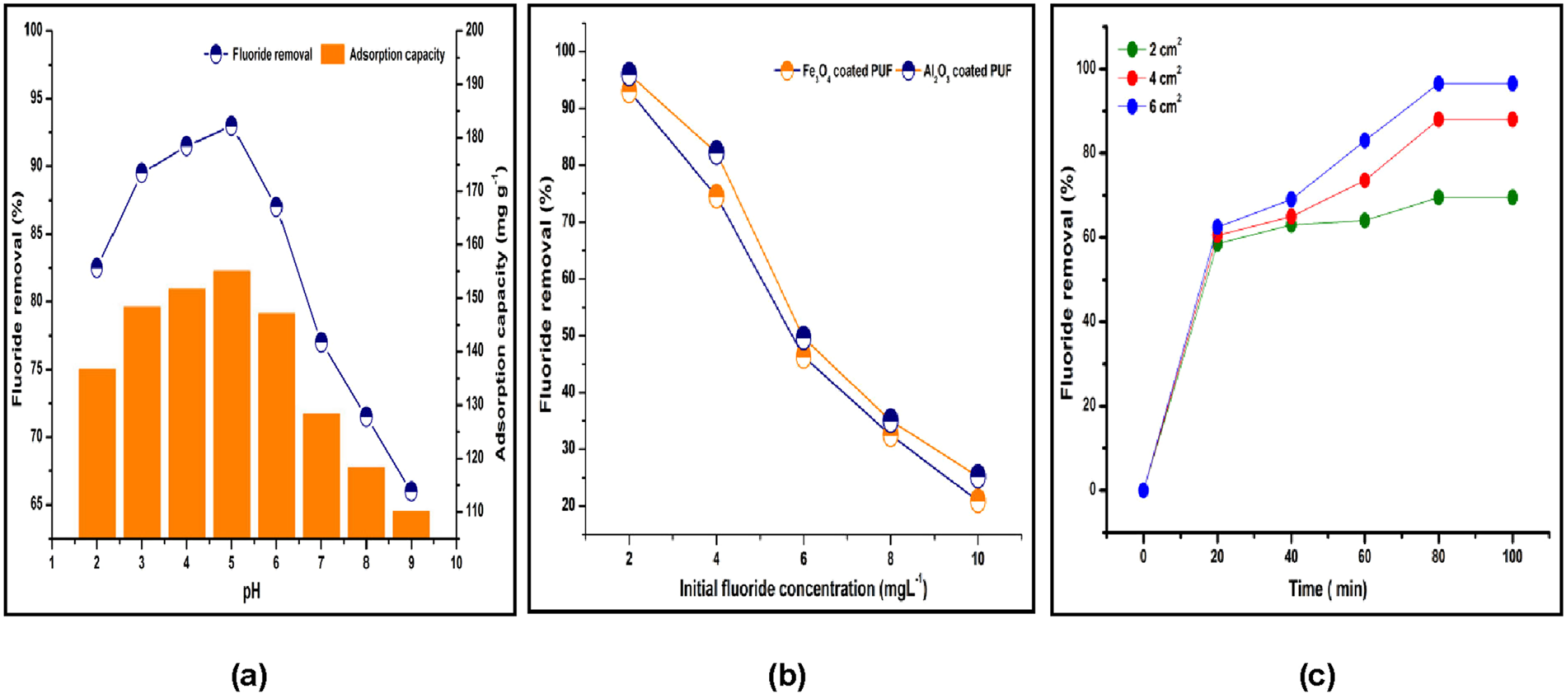
(**a**) Effect of pH (Time: 80 min, Foam size: 6 × 6 cm^2^ and Fe_3_O_4_ NPs-PUF), (**b**) Effect of Initial F concentration (Time: 80 min, Foam size: 6 × 6 cm^2^, pH: 5 for Fe_3_O_4_ NPs-PUF and 6 for Al_2_O_3_ NPs-PUF), (**c**) Effect of PUF size (pH: 6 for Al_2_O_3_ NPs-PUF, F concentration: 2 mg L^−1^).

The pH of the aqueous solution plays a noteworthy role in the F removal during both dip adsorption and batch studies. The surface charge of the mineral oxides is positive when pH value is below pH zero point charges (ZPC) and negatively charged when pH value is above the ZPC. The F adsorption by Fe_3_O_4_ NPs-PUF and Al_2_O_3_ NPs-PUF was observed to be strongly pH dependant. The percentage F removal increased with increasing pH up to 5 and 6 for Fe_3_O_4_ NPs-PUF and Al_2_O_3_ NPs-PUF, respectively. But F removal percentage is decreased in the pH range of 5.0–9.0 for Fe_3_O_4_ NPs-PUF and 6.0–9.0 for Al_2_O_3_ NPs-PUF. These results demonstrated the reduction in F removal upon enhancing the pH above 5 and 6 for Fe_3_O_4_ and Al_2_O_3_ NPs-PUF, respectively (Fig. 4a). In acidic pH conditions, the formation of hydrofluoric acid (HF) is responsible for the reduction of F adsorption. Under alkaline conditions, F removal declined because of the competition between F ions and hydroxyl ions for the active surface sites. In addition, the electrical repulsion among negatively charged adsorbent surface sites is probably responsible for the low absorbance.

#### Initial fluoride concentration and contact time effect

The adsorption of F ions reduced as the initial F concentration increased (Fig. 4b). The percent fluoride removal was found to be 93 and 96.3% for Fe_3_O_4_ NPs and Al_2_O_3_ NPs-PUF, respectively from the initial 2 mg L^−1^ F concentration, which further decreased to 20.9 and 25.2% for Fe_3_O_4_ NPs and Al_2_O_3_ NPs-PUF, respectively from the initial 10 mg L^−1^ F concentration at a contact time of 80 min (Fig. 4b). The variation in the percent F removal may be due to the decline in the number of available adsorption sites as they saturated at a excess F concentration. Adsorption behavior was studied as a function of contact time from 20 to 100 min with Al_2_O_3_ NPs-PUF size 6 × 6 cm^−1^ at pH 6 at 30 °C (Fig. 4c). It clear from the above results that the adsorption enhances with time and an equilibrium state is attained after a contact time of 80 min.

#### Effect of the varying PUF size on F adsorption

The effect of varying Al_2_O_3_ NPs-PUF sizes on F adsorption was evaluated using 2 mg L^−1^ initial F concentration and pH 6 (Fig. 4c). With the increase in the size of Al_2_O_3_ NPs-PUF from 2 to 6 cm^2^, the percent F removal also increased from 58.50 to 96.50%. The percent F removal by Fe_3_O_4_ NPs-PUF also increased up to 93% from initial 2 mg L^−1^ F concentration at pH 5. The presence of extra NPs on surface with increase in PUF size allowed efficient interaction resulting in enhanced interaction and overall percent F removal.

#### Fluoride concentration in tea infusions

The F levels are substantially found in all black, green and jasmine tea samples tested (Table 1). All tea products tested exceeded the permissible limit of 1.5 mg L^−1^ of F. F concentrations in leaf tea were considerably more than in bagged tea drinks. The F concentration in black tea was detected to be more than green and jasmine tea samples.

**Table 1:** F concentration in different tea infusions (R1 is Black tea sample 1, T1 is Black tea sample 2, L1 is Green tea sample 1, G1 is Green tea sample 2, J1 is Jasmine tea sample 1, J2 is Jasmine tea sample 2).

Tea type	Sample	Tea infusions with drinking water (mg L^−1^)	Tea infusions with deionized water (mg L^−1^)	F concentrations after defluoridation process in tea infusions using Al-PUF bag	F concentrations after defluoridation process in tea infusions using Fe-PUF bag
**Black tea**					
Leaf tea	R1	4.01	2.66	1.51	1.73
	T1	3.75	2.40	1.37	1.56
Bagged tea	R1	3.82	2.47	1.43	1.61
	T1	3.44	2.09	1.18	1.34
**Green tea**					
Leaf tea	L1	3.91	2.56	1.47	1.66
	G1	3.55	2.20	1.20	1.40
Bagged tea	L1	3.40	2.05	1.27	1.52
	G1	3.23	1.88	1.18	1.25
**Jasmine tea**					
Leaf tea	J1	3.69	2.34	1.42	1.49
Bagged tea	J2	3.41	2.06	1.23	1.41

#### Fluoride removal from tea infusions

The defluoridation studies were carried out by simply dipping the Al_2_O_3_-PUF and Fe_3_O_4_-PUF infusion bags in 100 ml of tea samples, respectively. The measured F concentration after the defluoridation process using Al_2_O_3_-PUF infusion bags was found to be under the permissible limit (1.5 mg L^−1^). However, use of Fe_3_O_4_-PUF infusion bags did show defluoridation but failed to show permissible F levels in all tea infusions tested (Table 1).

### Adsorption kinetics

The adsorption efficiency is illustrated using a variety of kinetic models. The adsorption kinetics was studied with pseudo-first-order and pseudo-second-order models. The data obtained was applied to pseudo-first-order and pseudo-second-order models to explain the adsorption kinetics of F ions on the Fe_3_O_4_ and Al_2_O_3_ NPs-PUF. The pseudo-first-order kinetic model is expressed by following eq. (2) ^37^.

2 $$\mathrm{log}({{\rm{q}}}_{{\rm{e}}}-{{\rm{q}}}_{{\rm{t}}})=\,\mathrm{log}\,{{\rm{q}}}_{{\rm{e}}}\mbox{--}({{\rm{k}}}_{1}/2.303){\rm{t}}$$

where q_t_ and q_e_ signify the quantities of F adsorbed (mg g^−1^) at time t and at equilibrium, respectively and k_1_ (h^−1^) is the first-order reaction rate constant. The pseudo-second-order reaction is expressed by following eq. (3) ^37^

3 $${\rm{t}}/{{\rm{q}}}_{{\rm{t}}}=1/{{\rm{k}}}_{2}{{{\rm{q}}}_{{\rm{e}}}}^{2}+{\rm{t}}/{{\rm{q}}}_{{\rm{e}}}$$

where k_2_ (mg g^−1^ h^−1^) is the pseudo-second-order rate constant for F adsorption.

The slope and intercept for both kinetic models were obtained by the linear kinetic plots, and kinetic parameters were determined as shown in Table 2. The obtained data demonstrated that the pseudo-second-order model fitted better for the adsorption study with highest correlation coefficient values (R^2^ = 0.996 and 0.997 for Al_2_O_3_ and Fe_3_O_4_ NPs-PUF) than pseudo-first-order model. Both the Al_2_O_3_ and Fe_3_O_4_ NPs-PUF materials followed pseudo-second-order kinetics reveling that the F ions uptake takes place by means of chemisorption processes.

**Table 2:** Kinetic parameters for F adsorption onto Al_2_O_3_ and Fe_3_O_4_ NPs-PUF at different initial F concentrations.

Fluoride initial concentration (mg L^−1^)						
Model	Parameters	2	4	6	8	10
Pseudo-first-order ^(Al)^	R^2^	0.912	0.993	0.923	0.946	0.890
	k_1_	0.023	0.011	0.036	0.009	0.034
Pseudo-first-order ^(Fe)^	R^2^	0.990	0.997	0.999	0.892	0.905
	k_1_	0.027	0.025	0.016	0.016	0.032
Pseudo-second- order^(Al)^	R^2^	0.968	0.962	0.992	0.955	0.996
	k_2_	0.0002	0.0004	0.0007	0.0010	0.0031
Pseudo-second-order ^(Fe)^	R^2^	0.988	0.997	0.988	0.974	0.994
	k_2_	0.0002	0.0010	0.0012	0.0011	0.0028

### Adsorption isotherm studies

To quantify the defluoridation capacity of NPs-PUF, three important isotherms were adopted. The experimental data obtained for the F concentration (2 mg L^−1^) at constant temperature and pH 6 and 5 for Al_2_O_3_ and Fe_3_O_4_ NPs-PUF were fitted to three commonly used isotherm models, such as Langmuir, Freundlich, and Temkin.

**Figure 5:**
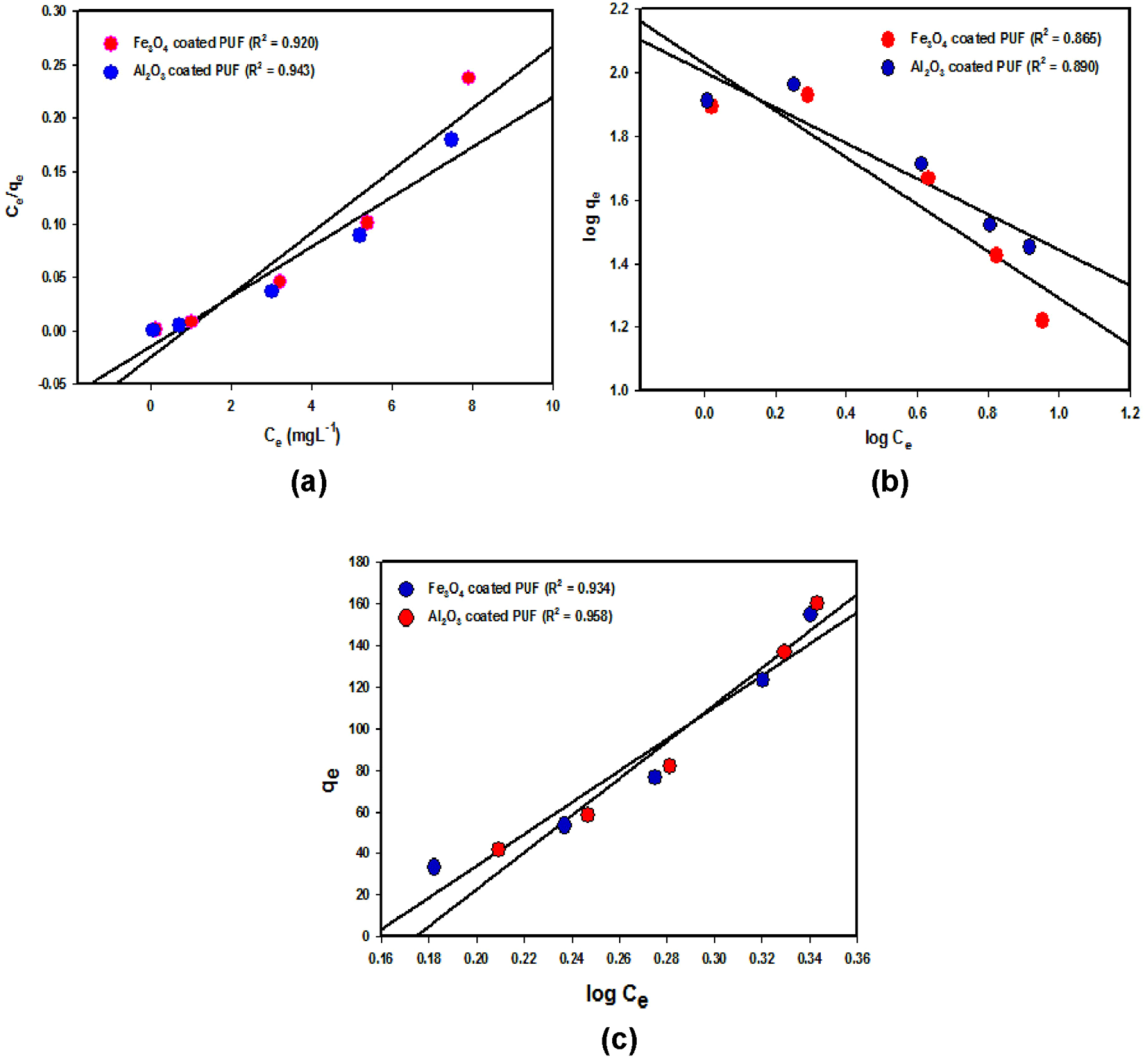
Adsorption isotherms on Al_2_O_3_ and Fe_3_O_4_ NPs-PUF. (**a**) Langmuir plot, (**b**) Freundlich plot and (**c**) Temkin plot.

The Langmuir isotherm describes the monolayer adsorption and is shown in the linear form the following eq. (4) ^37^:

4 $${{\rm{C}}}_{{\rm{e}}}/{{\rm{q}}}_{{\rm{e}}}={{\rm{C}}}_{{\rm{e}}}(1{/Q}^{0})+1{/Q}^{0}{\rm{b}}$$

where C_e_ is the equilibrium concentration of adsorbate (mg L^−1^), q_e_ is the amount of F adsorbed at equilibrium (mg g^−1^), Q° is the adsorption for a complete monolayer (mg g^−1^), and b is the Langmuir isotherm constant (L mg^−1^). Figure 5a shows that experimental data fitted well with the Langmuir isotherm, maximum adsorption capacity was found to be 43.47 and 34.48 mg g^−1^ for Al_2_O_3_ and Fe_3_O_4_ NPs-PUF with R^2^ values of 0.943 and 0.920, respectively. This result indicates the favorable adsorption of F on NPs-PUF.

The Freundlich isotherm shows adsorption on the heterogeneous surfaces and is expressed as shown in following eq. (5) ^37^:

5 $$\mathrm{log}\,{\rm{qe}}=\,\mathrm{log}\,{{\rm{k}}}_{{\rm{F}}}+(1/{\rm{n}})\mathrm{log}\,{{\rm{C}}}_{{\rm{e}}}$$

where k_F_ is the Freundlich isotherm constant (mg g^−1^) and n is the adsorption intensity. Figure 5b shows the linear plots of Freundlich isotherm of F ions adsorbed on the Al_2_O_3_ and Fe_3_O_4_ NPs-PUF. The values of n > 1 represent the favorable adsorption condition and the calculated n value in the present study was calculated to be 1.78 and 1.35 for Al_2_O_3_ and Fe_3_O_4_ NPs-PUF, respectively that proves the favorable isotherm.

The Temkin isotherm demonstrates as adsorbent-adsorbate interaction. A linear plot between q_e_ and log C_e_ demonstrates the Temkin isotherm as shown in Fig. 5c, which is defined by the following eq. (6) ^38^

6 $${{\rm{q}}}_{{\rm{e}}}={\rm{RT}}/{{\rm{b}}}_{{\rm{T}}}{\rm{In}}({{\rm{A}}}_{{\rm{T}}})+{\rm{RT}}/{{\rm{b}}}_{{\rm{T}}}{\rm{In}}({{\rm{C}}}_{{\rm{e}}})$$

for the Temkin isotherm, R is the ideal gas constant (8.31 J mol^−1^K^−1^), T is the absolute temperature (K), b_T_ is the Temkin isotherm constant (kJ mol^−1^) and A_T_ is the Temkin isotherm equilibrium binding constant (L g^−1^). The Temkin isotherm constant values b_T_ and A_T_ was calculated from the slope and intercept of the plot. The heat of adsorption values calculated using the Temkin model was 2.78 and 3.25 kJ mol^−1^ for Al_2_O_3_ and Fe_3_O_4_ NPs-PUF, respectively which correlated to the efficient adsorption of Al_2_O_3_ NPs-PUF followed by Fe_3_O_4_ NPs-PUF. This result is also consistent with the tested Langmuir isotherm model (Fig. 5a and c), which showed more favorable and maximum sorption capacity with Al_2_O_3_ NPs-PUF (43.47 mg g^−1^) than Fe_3_O_4_ NPs-PUF (34.48 mg g^−1^).

### Mechanism of defluoridation

The adsorption of F ions onto Fe_3_O_4_ and Al_2_O_3_ NPs-PUF is limited to the number of exchangeable hydroxyl groups, which is dependent on the surface area of the material.

**Figure 6:**
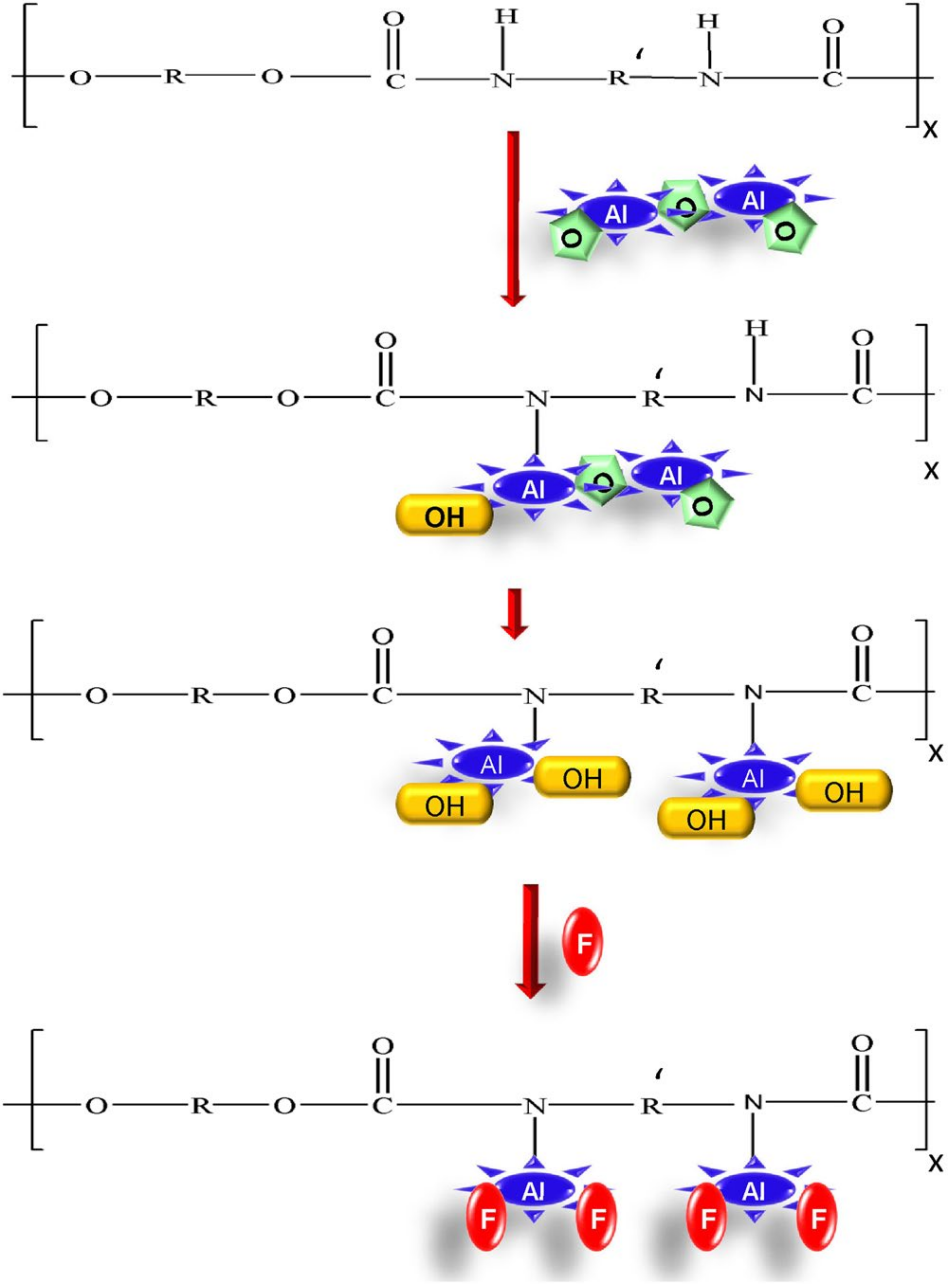
Proposed mechanism for the NPs impregnation on PUF and adsorption of F on NPs.

The FTIR spectrum of Fe_3_O_4_ and Al_2_O_3_ NPs-PUF after F adsorption shows that the hydroxyl groups are involved in the adsorption process. The decrease in peak intensity of hydroxyl groups at 3636 cm^−1^ in FTIR spectra of both Fe_3_O_4_ and Al_2_O_3_ NPs-PUF after F adsorption is a clear evidence that exchange in F ions occurred with hydroxyl ions (Fig. 3). This may be due to the similar ionic radius of the iso-electronic OH and F ions^39^. NPs impregnation and F adsorption mechanism on the PUF is illustrated as shown in Fig. 6. However, no further structural changes were observed in NPs-PUF samples after F adsorption that indicates the mechanism of F adsorption occurred through ion exchange process, i.e., OH were replaced by F ions in the adsorption process. The oxidation state of Al_2_O_3_ is higher, which enhances the affinity of Al_2_O_3_ for F as compared to Fe_3_O_4_. Another factor responsible for the high adsorption capacity of Al_2_O_3_ NPs-PUF is small size of Al_2_O_3_ as compared to Fe_3_O_4_^40^.

### Cost of NP-PUF bag

The materials required for the development of NPs-PUF pouches were easily available and inexpensive PUF, metal salt and empty tea bags. The estimated cost of each NPs-PUF is estimated to be US $0.05 (Table 3). This proves that the cost of a developed defluoridation technology is most affordable to the rural population and the areas in the resource limited settings.

**Table 3:** Detailed cost of one NPs-PUF filter bag.

Material	Amount used for one NP-PUF pouch	Rate of material (In Rupee)	Cost (In Rupee)
Polyurethane foam (PUF)	6 × 6 cm^2^	675/sq meter	2.43
Empty tea bag	1	120/250 bags	0.48
FeSO_4_	0.303 g	259/500 g	0.15
			Total = 3.06 Rupee/one FeNPs-PUF pouch
			US $0.05/one FeNPs-PUF pouch
Polyurethane foam (PUF)	6 × 6 cm^2^	675/sq meter	2.43
Empty tea bag	1	120/250 bags	0.48
(Al(NO_3_)_3_)	1 g	195/500 g	0.39
			Total = 3.30 Rupee/one AlNPs-PUF pouch
			US $0.05/one AlNPs-PUF pouch

## Conclusion

In this study, we reported on an inexpensive defluoridation technique that utilizes Fe_3_O_4_ and Al_2_O_3_ NPs that were green synthesized using jojoba defatted meal extract as reducing agent. These NPs were impregnated in PUF and fabricated tea infusion bags that were highly efficient in defluoridation of water and tea samples. F adsorption efficiencies of Al_2_O_3_ NPs-PUF and Fe_3_O_4_ NPs-PUF achieved were 43.47 and 34.48 mg g^−1^ at pH 6 and 5, respectively. The oxidation state of Al_2_O_3_ was higher, which enhanced the affinity of Al_2_O_3_ for F as compared to Fe_3_O_4_. Another factor responsible for the high adsorption capacity of Al_2_O_3_ NPs-PUF is the small size of Al_2_O_3_ NPs as compared with Fe_3_O_4_ NPs. The Langmuir and Temkin models provided better correlation with the experimental data than with Freundlich isotherm model. Kinetic analysis favored pseudo-second-order kinetic model revealing that the F ion uptake takes place by means of chemisorption processes on Fe_3_O_4_ NPs-PUF and Al_2_O_3_ NPs-PUF. The FTIR studies revealed that ion-exchange mechanism takes place between hydroxyl ions of NPs-PUF and F ions in samples. High F levels in tea infusion bags with black, green and jasmine tea were defluorinated to permissible F limits using Al_2_O_3_ NPs-PUF tea-bag like pouches. The developed technique reported in this study has the advantages of high F removal capacity, ease of operation, portability, portability, environmental friendliness and low cost and thus making this approach most desirable to resource limited settings, especially in the rural areas with high ground water F contaminations. We believe that present study provides an affordable solution for F removal for rural and poor population for health and safety.

## Methods

### Chemicals and materials

All chemicals and reagents used in this study were of analytical grade. Plant material was collected from AJORP (Association of Rajasthan for Jojoba Plantation and Research Project), Jaipur, Rajasthan (India). Ferrous sulfate (FeSO_4_) and aluminum nitrate (Al(NO_3_)_3_) precursor were obtained from Himedia, India. Sodium fluoride (NaF) was also supplied by Himedia, India and F stock solution (100 mg L^−1^) was prepared by adding NaF (0.0221 g) to millipore water (100 ml). Three common tea varieties, such as black, green and jasmine tea were procured from the local supermarket (Rajasthan, India). PUF and tea bag filter paper were procured from local suppliers and utilized after cleaning with millipore water. All experiments were carried out using Millipore ultrapure water.

### Preparation of *Simmondsia chinensis* (jojoba) defatted meal extract

Jojoba was selected for green synthesis of Fe and Al NPs because of its abundant cost-effective and easy availability as a waste byproduct of oil extraction process. Green synthesis of NPs was performed as previously reported, with slight variations^41^. Prior to NPs synthesis, defatted jojoba seed meal was obtained. For this, the seed’s were oven dried at 60 °C for 1 h and ground in a grinder. The resulting seeds powder was then refluxed in a soxhlet extractor for 24 h with in petroleum ether (1:6 w/v) for extracting oil. After oil extraction, the residual powder was termed as defatted jojoba meal and dried at room temperature for further use. Next, 10 g of defatted seed meal was added into 100 ml deionized water and boiled at 80 °C for 25 min. After cooling, the suspension obtained was filtered using Whatman’s No.1 filter paper and stored at 4 °C. The filtrate was further utilized as reducing and stabilizing agent for NPs synthesis.

### Fe_3_O_4_ and Al_2_O_3_ NPs synthesis

An aqueous solution of 0.01 M FeSO_4_ was freshly prepared for the reduction process. For the reduction of metal ions, 10 ml of jojoba seed meal extract was added in 20 ml of 0.01 M FeSO_4_ solution with constant stirring at 50 °C. Complete reduction of FeSO_4_ to Fe^+^ ions was confirmed by the color transformation from brown to black. The suspension was then centrifuged at 10,000 rpm for 15 min and the pellet obtained was repeatedly washed with millipore water and oven dried at 100 °C.

For Al_2_O_3_ NPs synthesis, aluminum nitrate (Al(NO_3_)_3_) was added into seed meal extract with 1:3 ratio (w/w) and allowed constant stirring at room temperature. The mixture obtained was microwave heated at 540 W for 7 min, which yield a yellow brown precipitate that was later centrifuged. The precipitate was washed with millipore water followed by methanol and dried at 100 °C in oven.

### Preparation of Fe_3_O_4_ and Al_2_O_3_ NPs - PUF

The impregnation of Fe_3_O_4_ and Al_2_O_3_ NPs onto the PUF was performed through dip adsorption method^42^. The NPs were suspended in 100 ml of distilled water for sonication. Then PUF were cut into a size of 2 × 2, 4 × 4 and 6 × 6 cm^2^ with 3 mm thickness for impregnation process. For impregnation, 2 × 2 cm^2^ size PUF was placed in 0.1 g NPs solution subjected to constant stirring at 200 rpm for 24 h at 30 °C in a shaker. Finally, the resulting NPs-PUF product was repeatedly washed with distilled water twice to remove un-anchored NPs on PUF. Thus obtained product (NPs-PUF) was dried at 80 °C in an oven. By increasing PUF sizes to 4 × 4 cm^2^, the NPs concentration was also appropriately increased to 0.2 g NPs concentration and thus maintained the NPs to PUF area ratio.

### Adsorption experiments

The F adsorption experiments were carried out using a series of F concentrations, such as 2, 4, 6, 8 and 10 mg L^−1^ with 50 ml solution and with different NPs-PUF sizes in flasks. The contact time was varied to 20, 40, 60, 80 and 100 min and the flasks were placed in shaker at 140 rpm. Effect of pH on the F adsorption was studied in pH range of 2–9. At the end of adsorption process, the residual F concentration was determined by fluoride ion meter (Thermo Scientific Orion, USA). The removal efficiency of the adsorbent was calculated using following eq. (7) ^37^:

7 $${\rm{Removal}}\,{\rm{efficiency}}\,\,( \% )={{\rm{C}}}_{{\rm{o}}}-{{\rm{C}}}_{{\rm{e}}}/{{\rm{C}}}_{{\rm{o}}}\times 100$$

where C_o_ and C_e_ (mg L^−1^) are the initial and equilibrium concentrations of F. The post adsorption NPs-PUF were removed and dried in a oven for further characterization using FESEM, EDX, and FTIR techniques.

### NPs-PUF tea bag

For F removal from tea, easy to use tea-bag like pouches containing Al_2_O_3_ NPs impregnated 6 × 6 cm^2^ PUF and tea bag filter paper were prepared. The tea bag filter paper was impregnated through dipping in NPs solution overnight at 30 °C. For F adsorption, the Al-PUF bag was dipped in 100 ml of prepared tea for 5 min and further analyzed for F removal. Similar tea-bag like pouches were also prepared using Fe_3_O_4_ NPs impregnated PUFs and the tea bag filter paper, further utilized for F remediation.

### Preparation of tea infusions

**Figure 7:**
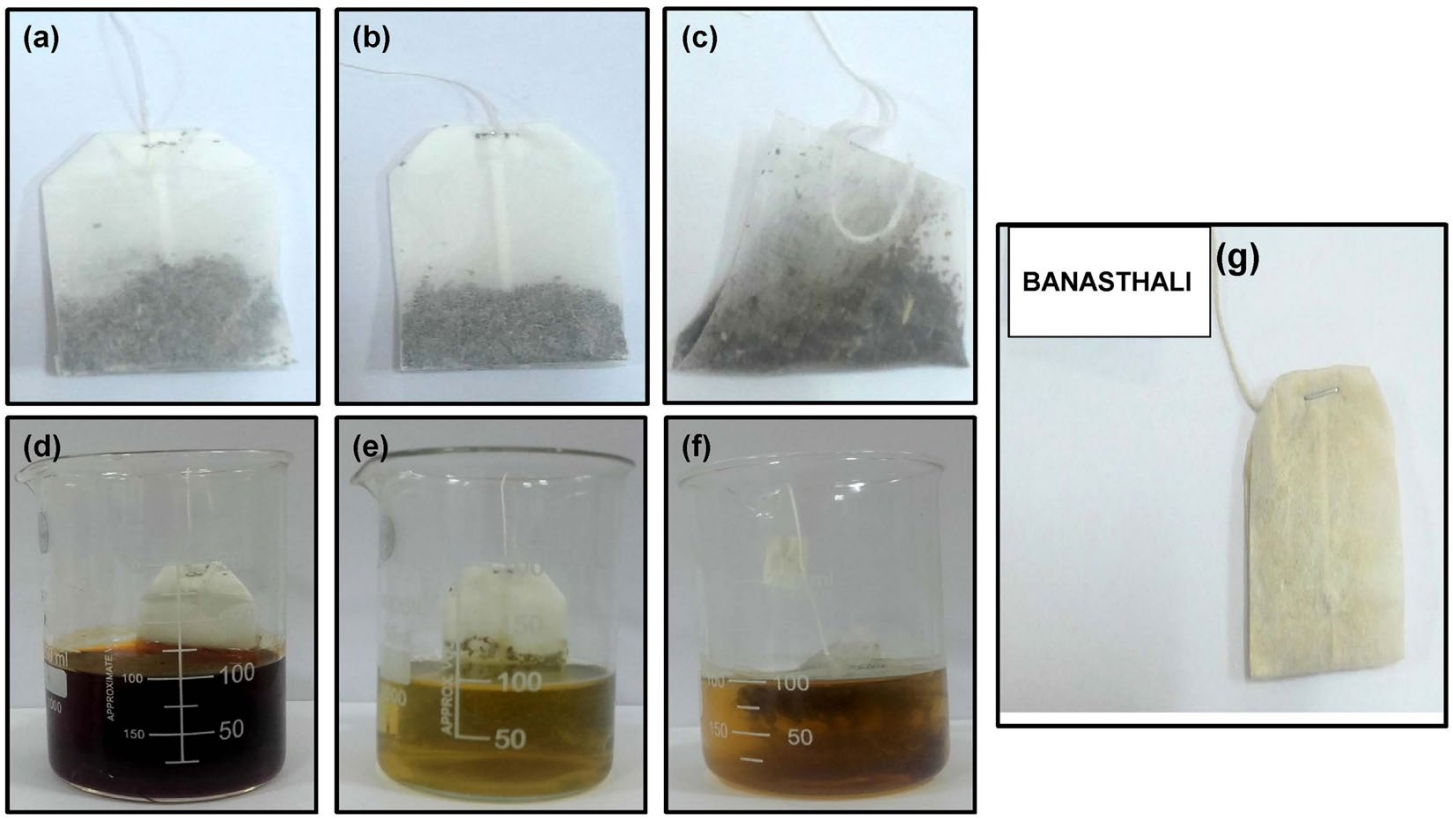
Tea bags of (**a**) Black, (**b**) Green and (**c**) Jasmine, Tea infusions prepared (100 ml) (**d**) Black, (**e**) Green and (**f**) Jasmine and (**g**) Novel designed Al-PUF tea bag.

Different tea bags, tea infusions and novel Al-PUF tea bag designed for F removal are shown in Fig. 7. The tap water was previously analyzed for F concentrations that were taken into consideration during the experiments. Each tea infusion was brewed for 2.5 min in 100 ml water at 95 to 98 °C, as the usual tea making time reported is 2 to 3 min^12^. After 2.5 min, tea infusions were filtered and allowed to cool for the F analysis.

#### Replication of the experiment

Each F adsorption experiment was conducted thrice and all the data share the average values of triplicate experiments.

### Characterization of adsorbent

The surface structure of Fe_3_O_4_ and Al_2_O_3_ NPs were observed by FESEM (MIRA3 TESCAN). Morphology of tea bag filter paper, pure PUF and NPs-PUF were characterized by FESEM. The elemental composition of both NPs was identified by EDX analysis. Also, the composition of NPs impregnated tea bag filter paper and NPs-PUF before and after F removal (post-adsorption) was analyzed. Phase identification and crystal structures of the NPs were characterized using an X-ray diffractometer (XRD Bruker D8 Discover). The surface charge of Fe_3_O_4_ and Al_2_O_3_ NPs was characterized by the zeta (ζ) potential. The isoelectric point of NPs was identified by titrating the *ζ*-potential over the pH range of 2–9. The pH of the solutions was adjusted by adding H_2_SO_4_ or NaOH. FTIR spectroscopy was carried out for Fe_3_O_4_ and Al_2_O_3_ NPs, NPs-PUF before and after F adsorption process for proposing an F adsorption mechanism. The thermal stability of the material was determined by Thermogravimetric analysis (TGA). The effect of NPs on the thermal properties of prepared NPs-PUF was analyzed.

### Data availability

No datasets were generated or analyzed during the current study.
